# Association of Demographic, Clinical, and Vaccination Characteristics with COVID-19 Viral Load Assessed by qRT-PCR

**DOI:** 10.34172/aim.2023.101

**Published:** 2023-12-01

**Authors:** Mohammadhossein Khorraminejad-Shirazi, Sara Sadat Nabavizadeh, Shiva Aminnia, Maryam Ahmadifar, Roham Borazjani, Erfan Sadeghi, Shabnam Izadpanah, Mina Heidari Esfahani, Maral Mokhtari, Ahmad Monabati

**Affiliations:** ^1^Department of Pathology, School of Medicine, Shiraz University of Medical Sciences, Shiraz, Iran; ^2^Student Research Committee, Shiraz University of Medical Sciences, Shiraz, Iran; ^3^Cell and Molecular Medicine Student Research Group, School of Medicine, Shiraz University of Medical Sciences, Shiraz, Iran; ^4^Otolaryngology Research Center, Department of Otolaryngology, Shiraz University of Medical Sciences, Shiraz, Iran; ^5^Trauma Research Center, Shahid Rajaee (Emtiaz) Trauma Hospital, Shiraz University of Medical Sciences, Shiraz, Iran; ^6^Research Consultation Center (RCC), Shiraz University of Medical Sciences, Shiraz, Iran; ^7^Shiraz Transplant Center, Abu-Ali Sina Hospital, Shiraz University of Medical Sciences, Shiraz, Iran; ^8^Raz Pathobiology and Genetic Laboratory, Shiraz, Iran; ^9^Pathology Department, Shahid Faghihi Hospital, Shiraz University of Medical Sciences, Shiraz, Iran

**Keywords:** COVID-19, SARS-CoV-2, Vaccine, Vaccine types, Viral load

## Abstract

**Background:**

The effect of vaccination on the SARS-CoV-2 baseline viral load and clearance during COVID-19 infection is debatable. This study aimed to assess the effects of demographic and vaccination characteristics on the viral load of SARS-CoV-2.

**Methods:**

We included the patients referred for outpatient SARS-CoV-2 qRT-PCR (reverse transcriptase quantitative polymerase chain reaction) test between July and September 2022. Cycle threshold (Ct) data were compared based on the demographic and vaccination characteristics. A generalized linear model was used to determine the factors associated with the SARS-CoV-2 PCR Ct value.

**Results:**

Of 657 participants, 390 (59.4%) were symptomatic and 308 (47.1%) were COVID-19 positive. Among 590 individuals with known vaccination status, 358 (60.6%) were booster vaccinated, 193 (32.6%) were fully vaccinated, 13 (2.2%) were partially vaccinated, and 26 (4.4%) were unvaccinated. Most vaccinated patients received inactivated vaccines (70.5%). The median Ct value was 20 [IQR: 18–23.75] with no significant difference between individuals with different vaccination statuses (*P* value = 0.182). There were significant differences in Ct value in terms of both symptom presence and onset (both *P* values < 0.001). Our regression model showed that inactivated vaccines (*P* value = 0.027), mRNA vaccines (*P* value = 0.037), and the presence and onset of symptoms (both *P* values < 0.001) were independent factors significantly associated with the viral load.

**Conclusion:**

The SARS-CoV-2 baseline viral load is unaffected by vaccination status, yet vaccination might accelerate viral clearance. Furthermore, we demonstrated that the presence and onset of symptoms are independent variables substantially associated with the patient’s viral load.

## Introduction

The rapid spread of SARS-CoV-2 infection led to a prompt effort to produce vaccines against the virus in a very short period. With multiple SARS-CoV-2 vaccines at hand, there is ongoing research on the efficacy of the vaccines in the context of emerging SARS-CoV-2 variants, especially breakthrough COVID-19 infections, and transmission since there are conflicting data regarding the influence of vaccination on the viral load of SARS-CoV-2.^1^

SARS-CoV-2 vaccination aims to protect individuals against severe COVID-19 and its sequels and, ideally, reduce onward transmission of SARS-CoV-2 in the human population. Currently available vaccines have varying levels of effectiveness against severe COVID-19, yet they do not provide sterilizing immunity; that is, breakthrough infections can occur in vaccinated individuals, and vaccinated individuals can shed virus upon exposure to SARS-CoV-2.^1-3^ However, COVID-19 vaccines might limit the magnitude of the viral load in cases of infection, thereby diminishing the onward transmission of SARS-CoV-2 from a vaccinated individual.^1,2^

 This study aimed to assess the effects of prior vaccination on baseline viral load levels after COVID-19 infection relative to unvaccinated individuals. We investigated whether the viral load from participants who had not been vaccinated would be higher compared with those who had been. We further aimed to evaluate the effect of vaccine doses and different vaccine types (inactivated vaccines, adenoviral vaccines, and mRNA vaccines) on viral load levels after infection. Also, we intended to evaluate the association of demographics, clinical, and vaccination characteristics with the viral loads of COVID-19.

## Materials and Methods

 We conducted a cross-sectional study on individuals referred for SARS-CoV-2 swab reverse transcriptase quantitative polymerase chain reaction (qRT-PCR) at the outpatient COVID-19 section of a tertiary pathobiology and molecular laboratory located in Shiraz, Fars, Iran. Data were collected retrospectively from July 2022 to September 2022. The Strengthening of the Reporting of Observational Studies in Epidemiology (STROBE) guidelines for cross-sectional studies were used for reporting and writing this study.

###  Study Population

 All patients referred to the outpatient COVID-19 section of a tertiary pathobiology and molecular laboratory located in Shiraz, Fars, Iran for SARS-CoV-2 swab nasopharyngeal PCR test from July 2022 to September 2022 were included in the research. Patients without a comprehensive registry of medical records were excluded. Moreover, participants who had received a dose of a SARS-CoV-2 vaccine less than 7 days before enrollment in the study, patients with previous positive history of PCR-proven COVID-19 infection, and referral samples from currently hospitalized patients were excluded.

###  Data Collection

Possible confounders on the cycle threshold (Ct) value were identified and chosen based on the previous studies that had evaluated the relationship of different factors with the COVID-19 viral load.^1,4-8^ The following factors were included in our study: gender, age, presence and onset of symptoms, comorbidities, type of vaccine, and vaccination status.

 Data were obtained from the laboratory registry database using a standardized data collection form. This data collection form included baseline information (i.e. sex and age), clinical manifestations (i.e. presence and onset), comorbidities (i.e. immunodeficiencies, type 2 diabetes mellitus, malignancies, and kidney transplantation), history of previous PCR-proven COVID-19 infection, type of vaccine (i.e. adenoviral, inactivated, mRNA-based), and vaccination status (i.e. unvaccinated, partially vaccinated, fully vaccinated, and booster vaccinated). Data on vaccination status and type were validated with national immunization records. Subsequently, qRT-PCR test results were recorded as Ct value from the laboratory database.

 We defined a participant as unvaccinated if they had not received any dose of the SARS-CoV-2 vaccine, partially vaccinated if they had received one vaccine dose, fully vaccinated if they had received two doses, and booster vaccinated if they had received three doses of vaccine. Also, clinical manifestations were defined as the presence of fever and chills, cough, sore throat, headache, fatigue, myalgia, loss of smell and taste, shortness of breath, and diarrhea. Different vaccine types were stratified as inactivated vaccines (i.e. BBIBP-CorV (Sinopharm), BBV152 COVAXIN (Bharat Biotech), and BIV1-CovIran (COVIran Barekat)), adenoviral vaccines (i.e. ChAdOx1-S/nCoV-19 (AZD1222, Oxford-AstraZeneca), rAd26-rAd5 (Gam-COVID-Vac, Sputnik V)), and mRNA vaccines (i.e. Moderna COVID-19 vaccine (mRNA-1273), Pfizer BioNTech (BNT162b2) COVID-19 vaccine).

 To estimate nasal viral RNA burden, we compared qRT-PCR Ct data from test-positive anterior nasal swab specimens. For each test, nucleic acid extraction was performed after the standard oro-nasopharyngeal swab specimen collection procedure. Molecular detection of SARS-CoV-2 was accomplished based on the E and S genes primer-probe sets, and the internal control were determined using the RNAse P (RP) gene. To clarify, for each specimen, we calculated Ct value for the RP internal control and both SARS-CoV-2 viral targets (E and S genes). If the RP Ct value < 40 and Ct value for each or both E and S gene were less than 40, then the result of the specimen was interpreted as positive. This enabled us to compare the Ct value of SARS-CoV-2 viral targets to the Ct value of the RP internal sample control to compensate for sampling and extraction quality. The same Real-Time PCR Detection System machine model (MIC, Biomolecular Systems) was used for all the tests.

###  Statistical Analysis 

Descriptive statistics were performed on demographic and clinical variables, and the data were compared between individuals who received different vaccine types and doses. Qualitative data were presented in frequency and percentages (%). However, due to the skewed distribution of quantitative variables, they were reported using the median, quartiles [Q1–Q3], and interquartile range (IQR). The Kolmogorov-Smirnov test was used to assess the normality assumption of Ct value data. Due to the non-normal distribution of the Ct value data, comparisons between groups were made using the Mann-Whitney U and Kruskal–Wallis tests for numerical variables and the Fisher or chi-square test when appropriate for categorical variables. All tests were performed in a two-tailed fashion, and a *P* value less than 0.05 was regarded as statistically significant.

Univariate generalized linear model was used to explore the association between identified variables and Ct value as the dependent variable among individuals tested positive for SARS-CoV-2 by PCR. Age was treated as a continuous variable, and symptom status was categorized into three classes (asymptomatic, symptomatic with symptom onset of less than 5 days, and symptomatic with symptom onset of equal to or more than 5 days). Subsequently, those variables with *P* value < 0.2 and important predictors in the univariate analysis were included in the multivariate model to determine which variables were independently associated with the Ct value. Results are expressed as regression coefficient and their associated 95% confidence intervals (CIs). Data analyses were performed using SPSS version 26.0 (IBM Corp., Armonk, NY).

## Results

###  Demographics and Clinical Characteristics of the Participants

During the study period (July to September 2022), 657 community-based participants who fulfilled the inclusion criteria were retrospectively recruited for our research. Among them, 299 (45.5%) individuals were female, and the median age was 38 [Q1–Q3: 33–47; range: 5–97]. SARS-CoV-2 was detected with qRT-PCR on nasopharyngeal swabs of 308 (47.1%) individuals. Except for 37 (6.3 %) participants, no one reported any comorbidities. Of 390 (59.4%) symptomatic participants, 317 (81.3%) had symptom onset of less than 5 days, and 73 (18.7%) had symptom onset equal to or more than 5 days. At the time of sampling, symptomatic individuals exhibited mild symptoms; however, the progression of the disease was unknown (Table 1).

**Table 1:** Demographic and Clinical Characteristics of the Study Participants, with Comparisons Between Different Vaccination Types and Statuses

**Variable **	**Total (N=657)**	Vaccination status^*^					Vaccine type^**^					
		**Booster (n=358)**	**Full (n=193)**	**Partial (n=13)**	**Unvaccinated (n=26)**	***P*** value^***^	**Inactivated (n=394)**	**Adenoviral (n=113)**	**mRNA (n=15)**	**Inactivated+Adenoviral (n=37)**	**Unvaccinated (n=26)**	***P*** value^***^
Gender; n (%)												
Male	358 (54.5)	205 (57.3)	102 (52.8)	4 (30.8)	10 (38.5)	0.071	202 (51.3)	72 (63.7)	7 (46.7)	25 (67.6)	10 (38.5)	0.024
Female	299 (45.5)	153 (42.7)	91 (47.2)	9 (96.2)	16 (61.5)		192 (48.7)	41 (36.3)	8 (53.3)	12 (32.4)	16 (61.5)	
Age (y); Median [Q1–Q3]	38 [33,47]	40 [34,51]	37 [30,44]	36 [26,41]	36 [31, 40]	< 0.001	38 [32,47]	40 [34,49]	45 [35,71]	36 [32,41]	36 [31, 40]	0.001
Symptom; n (%)												
Asymptomatic	267 (40.6)	116 (32.4)	66 (34.2)	7 (53.8)	16 (61.5)	0.010	137 (34.8)	27 (23.9)	4 (26.7)	17 (45.9)	16 (61.5)	0.002
Symptomatic	390 (59.4)	242 (67.7)	127 (65.8)	6 (46.2)	10 (38.5)		257 (65.2)	86 (76.1)	11 (73.3)	20 (54.1)	10 (38.5)	
Symptom onset; n (%)												
< 5 days	317 (81.3)	201 (83.1)	101 (79.5)	6 (100)	8 (80.0)	0.647	207 (80.5)	77 (89.5)	6 (54.5)	17 (85.0)	8 (80.0)	0.047
≥ 5 days	73 (18.7)	41 (16.9)	26 (20.5)	0 (0)	2 (20.0)		50 (19.5)	9 (10.5)	5 (45.5)	3 (15.0)	2(20.0)	
Comorbidities; n (%)	37 (6.3)	23 (6.5)	9 (4.8)	2 (15.4)	1 (4.2)	0.340	29 (7.5)	4 (3.6)	0 (0)	1 (2.7)	1 (4.2)	0.531
Positive COVID-19; n (%)	308 (47.1)	176 (49.4)	88 (45.6)	3 (23.1)	11 (42.3)	0.239	185 (47.2)	60 (53.1)	6 (40.0)	15 (40.5)	11 (42.3)	0.602

*Of these, the vaccination status of 67 patients was missing. **Of these, the vaccination type of 96 individuals was missing. ***P value equal to or less than 0.05 is considered statistically significant.

**Figure 1:**
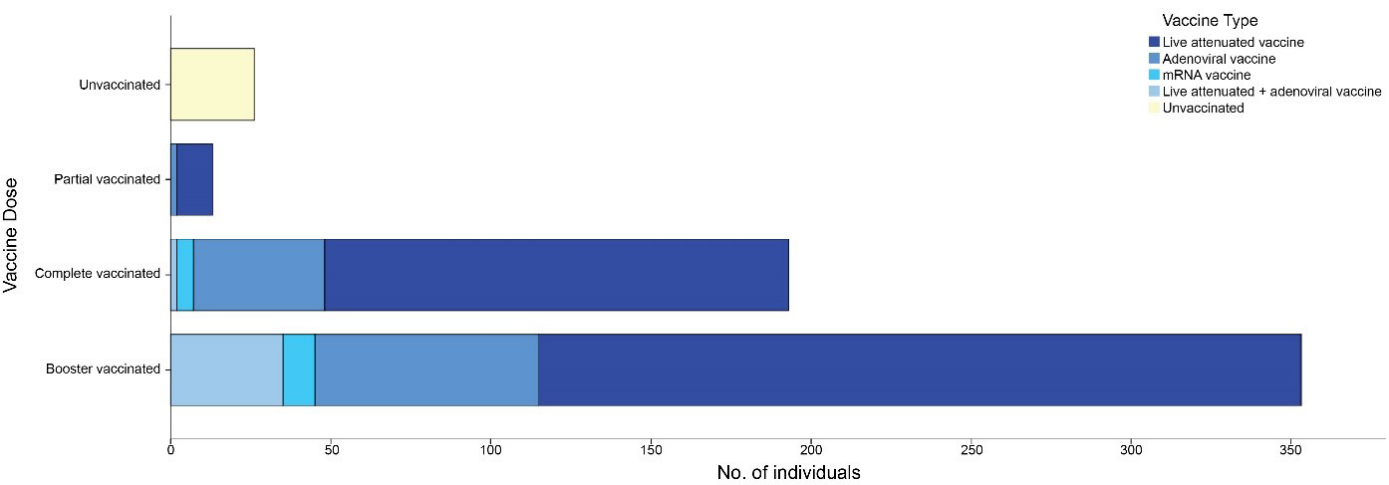
Stacked Bar Chart Denoting the Number of Individuals by Their Vaccination Characteristics

Among the 657 participants in our study, the vaccination status of 67 individuals was not documented; of the remaining subjects, 358 (60.6%) were booster vaccinated, 193 (32.6%) were fully vaccinated, 13 (2.2%) were partially vaccinated, and 26 (4.4%) were unvaccinated. Among vaccinated participants with a clear vaccine type (559 individuals), 394 (70.5%) received the inactivated vaccine, 113 (20.2%) received the adenoviral vaccine, 15 (2.7%) received the mRNA vaccine, and 37 (6.6%) received a combination of the adenoviral and inactivated vaccines. Figure 1 shows the distribution of the vaccination dose by vaccine type. The demographic and clinical features describing the different categories of the participants according to vaccination status and type are summarized in Table 1.

###  COVID-19 qRT-PCR Cycle Threshold Value

In the analysis of the Ct value of positive qRT-PCR samples, we found that the median Ct value was 20 [Q1–Q3: 18–23.75; range: 12–35], and there was no meaningful difference in the median peak viral load between the genders (*P* value = 0.067), and individuals with and without comorbidities (*P* value = 0.245). Of note, those suffering from diabetes mellitus had a Ct value of 18 [Q1–Q3: 16–30], and the participants who suffered from malignancies had a Ct value of 19 [Q1–Q3: 16.25–26.25] (with a lower peak Ct value, indicating a higher viral load). Furthermore, there were significant differences in Ct value in terms of both presence and onset of symptoms (both *P* values < 0.001). Moreover, among those who tested positive for COVID-19, fully and booster vaccinated symptomatic patients in whom the onset of symptoms was equal or more than 5 days showed significantly higher Ct values compared to those with symptom onset less than 5 days (*P* value = < 0.001) (Table 2).

**Table 2:** Comparison of the Cycle Threshold Value of Vaccinated and Unvaccinated Individuals Per Symptom onset Among Positive COVID-19 Samples

**Vaccination Status**	**Symptom Onset**		***P*** Value^*^
	**<5 Days**	**≥5 Days**	
Booster; Median [IQR]	19 [5]	27 [8]	< 0.001
Full; Median [IQR]	20 [3]	23 [9]	< 0.001
Unvaccinated; Median [IQR]	20 [7]	17 [2]	0.368

*P value equal to or less than 0.05 is considered statistically significant.

**Figure 2:**
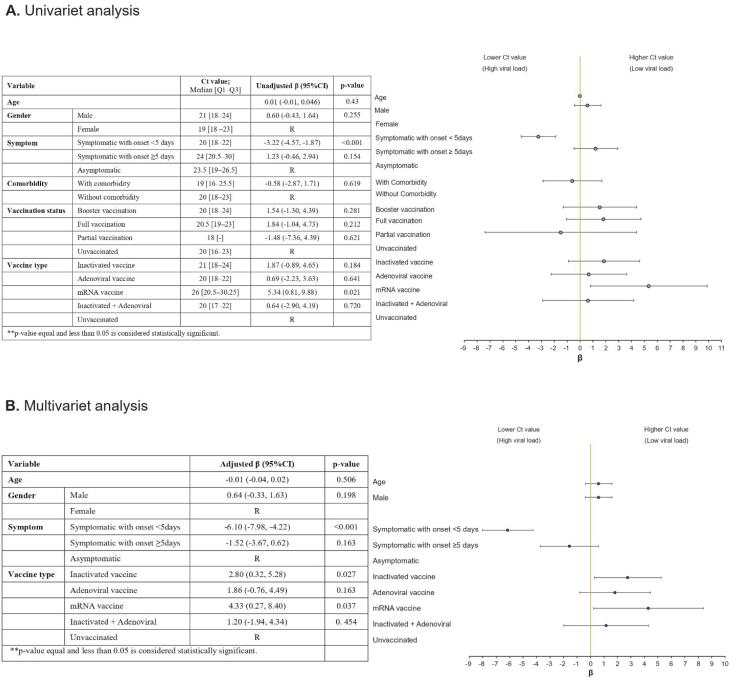
Univariate and Multivariate Generalized Linear Model Analysis of Independent Factors Associated with SARS-CoV-2 PCR Ct Value (R, reference level)

The median Ct value was comparable across the specimens from individuals with different vaccination statuses (booster vaccinated 20 [IQR: 6], fully vaccinated 20.5 [IQR: 4], partially vaccinated 18 [IQR: 0], and unvaccinated 20 [IQR: 7]; *P* value = 0.182). In terms of vaccine type, the Ct value was found to be higher in mRNA-vaccinated individuals compared to those who received other forms of vaccines although it did not reach statistical significance (*P* value = 0.086). The median [Q1-Q3] Ct values by different study variables are summarized in Figure 2A.

We evaluated our data based on univariate and multivariate generalized linear model, to assess the factors associated with the PCR Ct value (Figure 2). We did not observe any independent associations between age, gender, and the Ct value. Patients who were vaccinated with inactivated vaccines (regression coefficient, 2.80; 95% CI = (2.80, 5.28); *P* value = 0.027) and mRNA vaccines (regression coefficient, 4.33; 95% CI = (0.27, 8.40); *P* value = 0.037) had a higher Ct value compared to those with no history of vaccination. However, the Ct value was not significantly different for those who received adenoviral vaccines (regression coefficient, -1.52; 95% CI = (-3.67, 0.62); *P* value = 0.163) and inactivated + adenoviral vaccines (regression coefficient, 1.20; 95% CI = (-1.94, 4.34); *P* value = 0.454) compared to the unvaccinated ones. The symptom onset of less than 5 days was associated with a lower Ct value compared to asymptomatic patients (regression coefficient, -6.10; 95% CI = (-7.98, -4.22); *P* value < 0.001). Furthermore, individuals with the symptom onset equal to or more than 5 days had a higher Ct value compared with those whose symptom onset was less than 5 days (regression coefficient, 2.80; 95% CI = (2.80, 5.28); *P* value < 0.001) (Figure 2B).

## Discussion

We designed and performed this cross-sectional study to determine the effect of demographics, clinical features, and vaccination status on the viral load of SARS-CoV-2 infection and the association of these features with the PCR Ct value. It was shown that the PCR Ct values of outpatient participants diagnosed with COVID-19, as an indicator of viral load, were not statistically different between unvaccinated and vaccinated patients. In support of our data, several studies have found similar Ct values in breakthrough infections in vaccinated and unvaccinated patients.^8-12^ Additionally, we did not observe a significant difference between the Ct values of full vaccinated patients and those who had received the booster dose. This could be due to the fact that our participants were from the outpatient community-based population who had received their booster dose at different time points. As Levine-Tiefenbrun et al showed, the effect of vaccination on viral load declined 3 months after vaccination and disappeared after about 6 months, and could be restored with a booster dose.^8^

Interestingly, when compared to the asymptomatic participants, we observed that the symptomatic individuals showed notably higher viral loads. Other studies carried out by Blanquart et al and Dingemans et al have reported the same observation.^4,13^ Moreover, consistent with the literature,^1,6,11^ we found that infection in both complete and booster vaccinated individuals was characterized by faster clearance time than unvaccinated participants. This accelerated clearance time in vaccine recipients might potentially lead to reduced shedding of the virus and declined duration of infection.

We also compared the PCR Ct value in patients who had received different vaccine types. Although the Ct values did not differ statistically in patients who had received different vaccine types, the SARS-CoV-2 RNA load was found to be lower in mRNA-vaccinated individuals. Consistent with our findings, while Dingemans et al reported a higher proportion of breakthrough infection in individuals vaccinated with the adenoviral-based vaccines compared to the mRNA-based vaccines, they observed no significant difference when the type of the vaccine used and the RNA load were compared.^4^

In our study, we also evaluated the cases with high-risk comorbidities, i.e. patients with immunodeficiencies, type 2 diabetes mellitus, malignancies, and kidney transplantation. The median Ct value in this group was 19 [IQR: 10], with the median Ct value of the diabetic patients being 18 [IQR: 14], which was lower compared to the median Ct value of the general population of our study (20 [IQR: 5]); however, no statistical significance was obtained. Given that the decrease of 1 Ct unit is approximately equivalent to a two-fold increase in the number of viral loads per sample, this decrease in the Ct value of diabetic patients correlates with a more than four-fold rise in the viral particles. A retrospective multicenter cohort study carried out by Brosh-Nissimov et al^7^ showed that the higher proportion of patients with high-risk underlying diseases constructed the majority of breakthrough COVID-19 cases after full vaccination, and the higher SARS-CoV-2 viral loads were associated with poor outcomes. The findings of a retrospective cohort study by Lai et al^5^ depicted that booster vaccination was linked with a dramatic decrease in COVID-19 mortality rates among patients with multimorbidity. Since we evaluated a limited number of patients with comorbidities in our study, additional studies are required to assess the effect of vaccination on the viral loads of patients with comorbidities and allow the identification of high-risk individuals, who would require ongoing rigorous precautions and prophylactic measures.

 We also performed univariate and multivariate generalized linear models to identify the correlates of the independent variables with COVID-19 viral load. We found that the presence and onset of symptoms were independent factors associated with the PCR Ct value. Notably, we showed that the symptom onset less than 5 days significantly lowered the PCR Ct value. Additionally, the administration of inactivated vaccines and mRNA-based vaccines were independent factors associated with lower viral loads.

 Several limitations in our study must be acknowledged. First, we considered PCR Ct value as an indicator of viral load, and positive PCR results do not always correlate with a viable virus particle. Second, we did not evaluate which variant of SARS-CoV-2 was the dominant at the time of our sample collection. This might have affected the estimated viral load we evaluated in our study. Third, it is likely that there was a selection bias in the participants of the booster vaccinated group, such that high-risk individuals, like patients with high-risk underlying diseases and those with occupations that have more exposure to COVID-19, were probably among this group. Also, those who exhibited more symptoms were more likely to enroll in our study. Additionally, symptomatic vaccinated individuals in our research might be individuals with suboptimal vaccine reactions. Finally, when evaluating the vaccine type, there was only a limited number of individuals in the mRNA-vaccinated group. Thus, future studies with more participants are needed to better compare the effect of different vaccine types on the viral load.

## Conclusion

 Overall, the data provided in this cross-sectional study suggest that while vaccination does not affect the baseline viral load of SARS-CoV-2 infection, it might accelerate viral clearance. Also, we found no significant difference in the PCR Ct value when we comparing different vaccine platforms. Furthermore, we reported that the presence and onset of COVID-19-related symptoms were independent factors significantly associated with the viral load of the patients.
